# Exploring an Innovative Care Model and Telemonitoring for the Management of Patients With Complex Chronic Needs: Qualitative Description Study

**DOI:** 10.2196/15691

**Published:** 2020-03-06

**Authors:** Kayleigh Gordon, Carolyn Steele Gray, Katie N Dainty, Jane DeLacy, Patrick Ware, Emily Seto

**Affiliations:** 1 University of Toronto Toronto, ON Canada; 2 Centre for Global eHealth Innovation Techna Institute University Health Network Toronto, ON Canada; 3 Bridgepoint Collaboratory for Research and Innovation Lunenfeld-Tanenbaum Research Institute Sinai Health System Toronto, ON Canada; 4 North York General Hospital North York, ON Canada; 5 William Osler Health System Brampton, ON Canada

**Keywords:** models of care, complex patients, multimorbidity, telemonitoring

## Abstract

**Background:**

The growing number of patients with complex chronic conditions presents an urgent challenge across the Canadian health care system. Current care delivery models are overburdened, struggling to monitor and stabilize the complex needs of this growing patient population.

**Objective:**

This qualitative study aimed to explore the needs and perspectives of patients and members of the care team to inform the development of an innovative integrated model of care and the needs of telemonitoring (TM) for patients with complex chronic conditions. Furthermore, we explored how these needs could be successfully embedded to support this novel model of complex chronic care.

**Methods:**

A qualitative description design was utilized to conduct and analyze 29 semistructured interviews with patients (n=16) and care team members (CTM) (n=13) involved in developing the model of care in an ambulatory care facility in Southern Ontario. Participants were identified through purposive sampling. Two researchers performed an iterative thematic analysis using NVivo 12 (QSR International; Melbourne, Australia) to gain insights from examining multiple perspectives of different participants on complex chronic care needs.

**Results:**

The analysis revealed 3 themes and 13 subthemes, including the following: (1) adequate health care delivery remains challenging for patients with complex care needs, (2) insights into how to structure an integrated care model, and (3) opportunities for TM in an integrated model of care. Participants not only identified continued challenges in accessing and navigating care in a fragmented and disconnected delivery system but also identified the need for more self-management support. Patients and CTM described the structure of an integrated model of care, including the need for a clear referral and triage processes and composing a tight-knit circle of collaborating interdisciplinary providers led by a nurse practitioner (NP). Finally, opportunities for TM in an integrated model of care were identified, including increasing access and communication, the ability to monitor specific signs and symptoms, and building a clinical workflow around TM-enabled care.

**Conclusions:**

Despite entrenched health care service delivery models, a new model of care is acutely needed to care for patients with complex chronic needs (CCN). NPs are in a unique position to lead TM-enabled integrated models of care. TM can facilitate frequent and necessary monitoring of patients with CCN with more than one condition in integrated models of care.

## Introduction

### Background

Globally, approximately at least 1 in 3 adults suffer from multiple chronic conditions [1]. Nearly 40% of Americans have two to three chronic conditions, and 30% have four or more [2]. In Canada, it is estimated that 25% of the population has three or more chronic conditions [3]. In 2011-2012, 5% of the population accounted for nearly 65% of all health care spending in Canada, with heart failure (HF) and chronic obstructive pulmonary disease (COPD) being the diagnoses most responsible for acute care admissions [4]. Using the Ontario administrative data, another group found that high-cost users in the top 5% accounted for a greater admission rates, longer hospital stays, and alternate-level-of-care designations as compared with non–high-cost users [5]. Previous research demonstrates that adults who have high health care costs because of multimorbidity are also more likely to require more costly care in subsequent years [4,6].

### Health Care Delivery

Traditionally, health care delivery has focused on disease specialization [7,8] (eg, individual conditions) creating vertical integration of services and forming silos, often leaving behind the broader context of multiple risk factors and multiple chronic conditions. Current practice models are overburdened and often under-resourced to comprehensively and holistically address, monitor, and stabilize the complex health care needs of this high-cost, high-risk population. Comprehensive clinical management and patient care can be complicated by complex interacting medical and psychosocial issues. Adding to this care challenge, clinical guidelines are typically only available for individual conditions [9]. Thus, finding evidence-based holistic strategies, rather than a single-condition approach, to promote health and manage chronic conditions is essential to meet the needs of patients with complex chronic needs (CCN) [10]. The challenges in addressing the needs of patients with CCN have resulted in growing calls for major change to the delivery of clinical care [11,12]. Ontario has recently put forth a new provincial strategy heavily focused on integrated care, a fundamental shift from previous health care delivery approaches. However, the reality of integrating care still depends in part on the needs of patients, carers, providers, administrators, and researchers in the context of their individual experiences [7]. Exploring these perspectives in the context of informing a new model of care to manage these multiple and interacting needs is necessary to move forward on improved integrated services.

Previous research has concluded that the most effective interventions to improve the care of patients with CCN include a combination of multipronged care strategies [10,13]. Many of these strategies may benefit from support of information and communication technologies such as mobile health (mHealth) and telemonitoring (TM). TM allows patients to collect health information at home and for these data to be automatically sent to providers at a distant location. Thus, TM provides an opportunity to challenge traditional care delivery approaches and to leverage a patient’s ability to self-manage their condition(s) at home [14-18].

### Telemonitoring

Several systematic reviews have concluded that the use of TM can lead to improved clinical outcomes [14,18-21] and reduced costs for various chronic conditions [17,19,22]. For example, TM has been shown to reduce all-cause mortality in HF [20,23], improve hemoglobin A_1C_ in patients with diabetes [24,25], improve blood pressure in patients with hypertension [24], and reduce the frequency of respiratory exacerbations in COPD [22]. One study showed a significant decrease in depression and anxiety using a secure telephone and video service [25]. However, several large HF TM trials have reported mixed benefits and, in some cases, null results [26,27]. It is possible that the effect is not only because of the technology itself but also because of which conditions or lack of condition(s) are targeted in the research. For example, the majority of TM research is delivered in specialty care settings focused on a single condition, such as HF care delivered at a specialty clinic. Direct integration with practices and systems may also be to blame for inconsistent trial results [27]. Previous research has concluded that “interventions targeted either at specific combinations of common conditions or at specific problems for patients with multiple conditions, may be more effective than a single disease intervention approach” [28].

### Objectives

Unfortunately, there are limited examples of mHealth interventions developed specifically to support people with multiple CCN. This research presents a unique opportunity to contribute to this small but growing area of research to begin to develop a TM-enabled integrated care model informed by the needs of patients with CCN. Our study was guided by two research questions:

1. What are the needs and requirements of patients and their providers to develop an integrated model of care for patients with CCN?
2. How should TM be implemented and embedded in an integrated clinic model for patients with CCN?

## Methods

### Setting

Data collection was conducted at a large, ambulatory facility in Southern Ontario, Canada. This facility was recently funded to develop an integrated and comprehensive chronic disease management clinic to improve health care delivery and outcomes for patients with CCN. The goal of the clinic is to comprehensively address, stabilize, and clinically optimize short-term CCN using an integrated team-based approach. To support this goal, the intent was to study and implement TM in this clinic.

### Participants

Aligned with the vision of Margarete Sandelowski, a qualitative description design was utilized to incorporate multistakeholder perspectives into a new, unique model of care for patients with CCN. This approach was most appropriate for identifying varying needs and nuances of experience with which a TM system could be embedded and contribute to care delivery [28,29]. This approach was utilized to conduct qualitative semistructured interviews with patients and members of the care team [30,31]. As model development was in progress at the time of the interviews, researchers were interested in identifying how patient and provider needs could inform the integrated clinic model. Between June 2017 and September 2018, participants were recruited through purposive sampling during a soft launch period. All potential CTM consented to participate in this study. Team members including 2 physicians, 2 nurse practitioners (NPs), 2 registered nurses (RNs), 2 pharmacists, 1 social worker, and 4 administrators were recruited via email to ask if they would be interested in discussing their experiences in caring for patients with CCN. Interviews were conducted in person onsite or over the telephone, based on the preference of the care team member participant. Informed and written consent was obtained before all interviews. Patient participants were eligible to participate if they were at least 18 years old, were diagnosed with HF or diabetes as well as at least one other chronic condition, and could communicate in English [32,33]. The other conditions included hypertension, COPD, obesity, Parkinson disease, asthma, dyslipidemia, anemia, coronary artery disease, chronic kidney disease, stroke, osteoporosis, arthritis, gout, depression, or anxiety. Eligibility criteria for the model (outside of this inclusion criteria) included patients with multiple comorbidities, one or more inpatient hospitalization or two or more emergency visits within the last 6 months related to the chronic conditions, and a Length of stay, Acuity of admission, Comorbidities, Emergency department visits (LACE) score greater than 5 Exclusion criteria for this clinic included diagnoses of chronic pain, cirrhosis, dialysis, transplant, and severe dementia and long-term care residents. Patients were identified by NPs and recruited by the study coordinator (KG). Informed and written consent was obtained by the study coordinator. In some cases, a family member or caregiver signed the consent form if the patient was unable to sign independently. Given the role of caregivers in managing the needs of patients with CCN, the caregiver was invited to stay or asked to leave during the interview based on the patient’s preference. Caregivers were not asked any specific questions during the interview. None of the patient or team member participants had ever utilized a monitoring technology or system.

All research activities were undertaken with ethics approval from the William Osler Office of Research Ethics (number 17-0008) and the University of Toronto Research Ethics Board (number 34581).

### Qualitative Interviews

A semistructured interview guide was developed to facilitate an open and abductive discussion around CCN (Multimedia Appendix 1). The guide consisted of open-ended questions and exploratory prompts. At the beginning of each interview, the interviewer explained the objectives of the study (“to better understand the needs of patients with CCN and the needs of their CTM”). Prompts were used to dive deeper into the needs of the participants based on their experiences [29]. Team members were similarly asked to describe their perspectives related to managing patients with CCN in the context of the care model. Four care team interviews were conducted just before the soft launch of the model, and all patient interviews were conducted after the launch of the model. Participants were given the opportunity to ask questions at any time. Initially, 10 interviews were planned; however, additional interviews were conducted in both groups until data saturation was reached, meaning no new relevant information was discovered [34], and the sample size sufficiently answered the research questions [29]. Patient interviews lasted on average between 22 and 45 min, and care team interviews lasted between 28 min and just over 1 hour. The study coordinator had no relationship to the participants. All interviews were audio-taped and professionally transcribed verbatim.

### Analysis

A qualitative description approach was used to thematically analyze the interview transcripts. Data were analyzed by two researchers (KG and PW). The two authors read and reread the transcriptions to become familiar with the data and to pull together detailed analysis of their contents. Initial codes were identified and synthesized into categories and themes. We coded questions around the needs, the model, and TM (deductive), but the subthemes emerging from these areas emerged from the data inductively. NVivo software version 12 was used to organize and inductively form a coding matrix based on the data. Both researchers independently coded the transcripts and met to discuss the findings. Analysis was iterative over a prolonged period of time (>1 year) as codes were reviewed and discussed until a consensus was reached. The transcripts were considered all together by looking at the data within each thematic grouping across participants. No follow-up interviews were conducted in this study.

Member checking is a technique used to explore the credibility and resonance of the results with the participants [35]. A member check of the synthesized analyzed codebook was undertaken by sharing codes, categories, and themes with five team member participants after the initial interviews [35]. These participants provided in-person feedback after the soft launch of the model.

The lead author (KG) is an RN who has worked extensively with patients with CCN. The second coder (PW) is trained as qualitative researcher with a background in implementation. Throughout the process, we acknowledged our experiences by creating memo logs and discussing our interpretations of the data, particularly during coding. This provided an important opportunity to reflect upon our implicit assumptions and potential biases during the analysis and writing process.

## Results

### Overview

The needs and perspectives of patients (7 female and 9 male) and team members (12 female and 1 male) were organized into three core themes with relevant subthemes.

### Theme 1: Adequate Health Care Delivery Remains Challenging for Patients With Complex Care Needs

Patients and care team participants described the management of their needs as, at times, being challenging and complex. Patients with CCN experience challenges such as multiple providers, multiple care sites, accessing comprehensive services, navigating care services, and utilizing self-management tools. Both participant groups suggested that these challenges are not well aligned within the current health care delivery model. Patients with CCN frequently cross care specialties and care sectors. Team members echoed the experiences of the patients in terms of the challenges in providing comprehensive care to this patient population with CCN.

#### Continued Lack of Access to Care Services

Patients overwhelmingly described a lack of timely access to important health care services when needed, including family physicians and specialists in the current care model. As one patient noted:

Sometimes it takes up to a week to see my family doctor.

Patient (PT) 05

I’ve had diabetes for quite a number of years. I was diagnosed with heart disease, and blood pressure problems two years ago... sometimes, you know, you want to get a doctor’s appointment and you can’t get in.

PT 04

Team members similarly experienced difficulties in scheduling sudden needs-related appointments in family medicine, for example, during an acute exacerbation of their conditions:

Unless it’s absolutely urgent you can’t get an appointment very quickly and it at least takes a month to get an appointment.

CTM 01

Even when patients are able to access an appointment quickly (whether with a family physician or a specialist), team members acknowledge that the time allotted per patient in a visit (typically 5-15 min) is insufficient to comprehensively address, assess, and treat a patient with multiple, and often interacting, complex needs. They acknowledged that these limitations place an added burden on the patient and reliance on self-management.

#### Challenges Navigating a Fragmented System

Several participants described their perceptions of complexity as contributing to their experiences of care fragmentation. For example, multimorbidity, such as the number or combination of conditions, was mentioned as a contributing factor to complexity, leading at times to fragmentation. As one patient stated:

It feels like all the health problems join each other.

PT 01

Other needs, often unrelated to any one specific medical condition such as sleep apnea, anxiety, or depression, were not typically addressed in family medicine or specialty care, thus contributing to complexity and splintered care experiences. Navigating through a fragmented delivery system, even when timely access to individual services is available, seems to remain a significant challenge without a clear point of contact. According to CTM, communication is particularly poor when coordinating between multiple providers and managing many medications:

I think part of the problem is they’ve got prescriptions from multiple providers and that’s where the difficulty lies... a cardiologist will say this, the endocrinologist will say that, if they’re followed by nephrology they might have a completely different set of instructions and so how do you coordinate the specialists’ care plans or the specialists’ treatment plans and make is [sense] for a patient?

CTM 02

Contributing to a sense of fragmentation is the gap in communication between patients and their inner circle in between visits:

Sometimes we just pawn patients off and we really don’t know what happened because there is no communication until we see them for the next visit two or three months later.

CTM 08

Therefore, the lack of face-to-face time with providers, in addition to fragmented communication, can lead to frustrating and unfruitful interactions from the perspective of some of the patients interviewed:

Sometimes if you ask a lot of questions, it’s almost like they lose patience with you. The heart doctor, sometimes I wonder if he’s frustrated [of] me asking these questions, and it’s that I get confused because I think, well, you’re my heart specialist. Shouldn’t [I] be getting all this information?

PT 05

#### Lack of Technology Interconnectedness

The continued lack of interconnectivity within monitoring technologies was identified by patients as particularly challenging. One story provided by a patient describes how their monitoring device could only be interpreted at one hospital with no ability to share that information to their wider care network. Another patient discussed how his glucometer could not send readings automatically to his providers. Abnormal readings were only identified when he brought the device in at the next appointment:

They have to wait until I get the meter back [to the clinicians]. As far as having sort of a running inventory of what’s going on, they have no way of knowing. They’re sort of out in the cold, waiting for me to come along and present them with a unit that says, hey, you missed this one [a reading], you missed that one.

PT 06

#### Desire for Self-Management Tools in a Provider-Centric System

Patients expressed that they lacked access to tools such as educational resources or technologies needed to independently self-manage their care needs. CTM indicated that having time to discuss a patient’s self-management goals and barriers would enable a foundation to build self-management education in an integrated patient-centered care model. When describing the available tools and services for patients with CCN, several patients felt that their current providers lacked treatment options to meet their needs. In many cases, interacting and competing symptoms were too overwhelming to coordinate a lasting solution:

My family doctor...during my last visit, stood in front of me and said, “well, I don’t know what I can do for you anymore, I’ve run out of alternatives,” which made me feel that I needed something more, some whole evaluation of what was actually going on with me.

PT 06

Although CTM value the notion of patient-centered care, they acknowledge that the current model of care remains too provider-centric vs patient-centric to support patients in the engagement of self-management:

The point of care around collaborative and self-care planning... we really want to move away from being the clinical expert and being provider centric. We want to really be saying, our engagement with you as a patient or as a family member is about partnership and I think that’s critical when it comes to self-management.

CTM 07

### Theme 2: Insights Into Structuring an Integrated Model of Care

As part of the needs assessment, participants were asked specifically about how they could envision a care model that would meet their needs. In particular, researchers were interested in understanding how patients with CCN could be identified and referred into this type of care model, which type of providers should be a part of an integrated team, and how long should patients be followed up in this unique care model.

#### Need for a Structured Referral and Triage Process

Team members felt strongly that a structured identification, referral, and triage process should be based on the patient’s complex care needs, the number of diagnosed conditions, and recent emergency department visits/hospitalizations. Team members described the types of patients who would benefit from this new integrated model of care, such as high-risk patients streamlined for early discharge and patients who had at least two previous visits to the emergency department in the past 6 months:

Looking at patients who are readmitted within 28 days to hospital, recurring admissions, recurring ED visits. We’re going to try to focus on those people who obviously need the help and figure out a way to get referrals from those, tracking those people.

CTM 01

Working with primary care, local emergency departments, and urgent care facilities within the care network was also important in identifying high-risk patients in need of immediate stabilization but not necessarily hospitalization. Several participants acknowledged that there was no pathway for patients with CCN to obtain frequent monitoring and stabilization except going back to the family physician:

I think we’re going to see the patients for whom the primary care provider just feels, I can’t make all the connections. I need to send them somewhere for stabilization then I want them to come back with a care plan that makes sense for me.

CTM 02

#### Creating a Circle of Care

Both patients and CTM spoke of the potential advantages of creating a circle of care around the patient in this new care model. Specifically, a relatively tight-knit circle of collaborating multidisciplinary providers (ideally in one physical location) was described as an optimal model:

A lot of the patients can be managed by the RN. So, the NP will stabilize them, establish an initial plan of care and then the RNs could be almost seen to be as care navigators, so bringing in the dietician, social worker, kinesiologist when it’s needed but I see that team developing an initial care plan for the patient. Having them come back maybe every two to three months, seeing if we can stabilize them but eventually they’d be discharged back to primary care.

CTM 02

Several CTM strongly felt that a multidisciplinary team–based approach would most support the unique needs of patients with CCN. When probed, they identified specific roles such as nursing, social work, pharmacy, and dietary services as being important roles to include in creating an integrated team-based clinic for patients with CCN. Specialists, such as cardiologists and internists, were seen as being necessary peripheral resources that they could utilize when clinically necessary, outside of the routine or immediate integrated care management in this model. These specialists were viewed as critical contributors when the complex needs of the patient are in more advanced states or out of the NP’s scope of practice. Participants viewed family physicians as outside of this internal integrated model of care, so they could organize their needs in one place, in the period between hospitalization and repatriation to family medicine:

Even if you don’t think you need it, I think when you get that complex there’s usually social work type issues depending on the social status of the patient...[Specialists] to me are sort of the outer circle of care.

CTM 02

Patient and provider participants unanimously felt confident in the role of an NP as the central clinical and coordinating provider. Team members noted that the role of the NP could be as a gatekeeper coordinating complex care between family medicine and specialty care services. This circle of care was described as not to replace the patients’ current providers but rather to bring together and better coordinate services around the patients’ complex needs through more frequent monitoring:

We are trying to avoid multiple people and multiple visits...I see the gatekeeper as being the NP [at the] point of entry to understand all the specialty needs of the patient... and then the team all having a chance to review that patient’s care.

CTM 12

It’s good, the attention [from the NP]—normally I feel like I have to, like, just view and try to get in the most important stuff down, you know, just... health stuff, but it’s that whole thing of being on-edge for not having time to talk about something [my needs].

PT 16

As the implementation was underway, we were interested if there were other providers that could contribute expertise to this novel care model. In several cases, a respiratory therapist was identified as a role that could greatly contribute to the patient’s care, particularly for patients with COPD and asthma. Family members were also identified as critical members in this new model of care for both participant groups.

#### Length of Clinic Enrollment

Overall, the team members felt that a needs-based approach should be taken when determining how long patients should be enrolled in this unique care model for complex management:

It depends on the patient need. We are thinking 6 months. But with the complex cases, elderly, multiple complex things going on with the patients we would extend it, probably 12-18 months.

CTM 12

When probed, 6 to 12 months was seen as an estimated average period for stabilization of complex needs and to repatriate care back to the family physician:

It’s not a forever clinic. So you stabilize and try and get a solid plan in place, and then they’re discharged... I personally think six months might be a good goal... hopefully you can get a plan that will stabilize.

CTM 04

Overall, most patients were unsure of the right amount of time for optimal stabilization, but some of them felt strongly that it should be based on their individual needs at the time.

### Theme 3: Opportunities for Telemonitoring in an Integrated Model of Care

Participants were interested in the potential opportunities for TM in an integrated care model for patients with CCN. The ability to utilize TM to enable communication within the circle of care and between patients while improving access to care through frequent monitoring was a strong incentive. Participants described an alignment with monitoring physiological metrics already routinely measured as part of self-care. For example, a patient with HF is typically instructed to record daily weight and/or blood pressure readings as part of their care plan. TM could also present new opportunities to monitor more than one condition from a distance and facilitate face-to-face video visits, where more context around a specific reading could be provided and communicated to the team. However, team members felt a new clinical workflow would be required to facilitate this TM-enabled triage and workflow.

#### Enabling Communication Within the Circle of Care

The ability to communicate within the circle of care (eg, on ongoing patient monitoring, clinical assessment, patient evaluation, and care improvement) was identified as one of the strongest potential incentives for using a TM system by team members. As one care team participant noted, the ability to see a daily trend could inform clinical discussions as an integrated team:

[The ability] to see if there’s a trend and letting the team know, okay maybe we should sit down and talk about this patient, I think we need to bring them in sooner...[It] allows us to more closely check their status to see if whether or not we should bring them in earlier or should consider admitting the patient.

CTM 09

#### Increasing Access to Care Through Frequent Monitoring

Not all participants were aware of TM and even fewer had experiences with this type of technology. However, both groups discussed the potential opportunities of TM for patients with CCN. Several participants noted the opportunities for more frequent monitoring such as an opportunity for early identification of HF patient decompensation and coordination of care within the integrated team. One team member even postulated how improving access to care services utilizing TM could reduce avoidable visits to the emergency department or hospital:

I think it enhances accessibility to the clinic and patients with chronic medical problems are always unpredictable. You never know when an event is going to take place so I think that if they had accessibility...but you can prevent medical crises, you can prevent presentations to emergency... if they could access because sometimes it’s just a quick question or sometimes it’s more serious.

CTM 03

#### Important Telemonitoring Features

Patients identified how TM could be a useful technology for monitoring metrics they already routinely measure such as blood pressure, weight, and blood sugar. Patients also expressed an interest in the ability to monitor symptoms such as difficulty in breathing, sleep patterns, and anxiety. Many existing TM systems alert clinicians if the parameters fall outside a target or normal range, and participants had different preferences with respect to the modality for receiving these alerts (eg, email, text, and call):

For me, it [TM] could help, it would really help me to manage my conditions better, my diabetes readings, my cholesterol readings, my high blood pressure. It probably would alert me when things are coming pretty close to the edge, you know? I think I’d benefit from it.

PT 01

Similarly, team members described the perceived usefulness of monitoring specific symptom trends and mental health conditions (eg, depression and anxiety) known to be prevalent in this population:

We also see a lot of mental health and depression is very common with patients with chronic disease and it affects the patient's ability to self-manage...so having some sort of help mental health wise, I think, this [telemonitoring] would really help. I would say the majority of our patients do have depression and some people have some complex mental health issues.

CTM 01

In addition to an ability to track condition-specific parameters, team members identified the need for features that facilitate communication processes and care coordination. For example, several team members noted that patients would benefit from a comment section or face-to-face video, where more context around a specific reading could be provided and communicated to the team:

Having room for comments to explain if they are having a symptom, and they can explain what’s new or what’s different so that we could figure out why is this happening at this time... These are just values and they mean nothing without the context.

CTM 10

#### Building a Clinical Workflow Around Telemonitoring-Enabled Triage

Providers felt that clinical notification and alerts could be managed by an RN. Several team members discussed the opportunity to utilize a triage process similar to that of routine clinical practice, for example, triaging calls or TM alerts as clinically necessary to the most appropriate clinical provider (RN to the NP and NP to the physician) on a case-by-case basis:

I actually think the RN should be alerted—and she can triage what the situation is and depending on the situation she can notify the NP.

CTM 01

Team members felt it was important to identify explicit criteria and create a formal triage process for alerts within the workflow in advance of implementing a TM system for it to effectively support this integrated model of care. In particular, they felt it is within the scope of practice of an RN to act on the information provided by the patient through TM. Participants also felt that operating procedures that outline these explicit criteria should be based on the context of the clinic and an individual’s workload. For example, participants questioned the responsibility of monitoring on evenings and weekends:

There needs to be clear criteria for why you would notify the physician. I’m speaking now as a physician, so that you know up front the reasons and you’ve agreed with the reasons and they’re acceptable to you. I think the way that doctors triage in their mind is different than let’s say the way that the pharmacist might triage... or the dietician, and so the team has to decide in advance when different members are going to be notified and about what they’re going to be notified.

CTM 13

#### Potential Challenges

Participants identified several potential challenges to utilizing TM in this population, including tech-savviness and physical constraints (eg, vision impairments and manual dexterity concerns). Language was also identified as a potential challenge to consider when implementing TM in an integrated model of care for patients with CCN. Both participant groups suggested that language translations should be tailored to the population.

#### Promoting Self-Management

Finally, providers spoke of how TM might influence a patient’s accountability in managing their own care by connecting more frequently with their care team:

I think for sure the connection to the clinician, that virtual connection is invaluable to the patient. Not only from feeling secure and feeling that someone’s there to help...I think just that information that's being collected, that helps the patient understand their disease and their response to disease as well. They become more knowledgeable about what’s going on with them.

CTM 04

Some patients suggested it would help them manage their conditions better while hopefully avoiding unnecessary exacerbations, but none of them specifically mentioned accountability:

For me, it [TM] could help, it would really help me to manage my conditions better, my diabetes readings, my cholesterol readings, my high blood pressure. It probably would alert me when things are coming pretty close to the edge, you know? so I think I’d benefit from it.

PT 01

However, participants maintained that although the need for engagement is important in self-management, TM may not be for everyone. Patients suggested that those familiar with technology may be more inclined to participate in a TM program. Several patients also suggested that regardless of their current conditions, those familiar with technology may adhere to taking TM measurements differently.

## Discussion 

### Principal Findings

This paper provides an overview of the needs of patients and clinicians in developing an integrated care model for patients with CCN and the needs of TM. Study findings revealed that significant gaps remain in meeting the needs of patients with CCN in current health care delivery practice [36,37]. Patients continue to navigate a fragmented care system [36] between primary care and siloed specialty care, creating challenges to timely access and care management. Previous literature has well documented this *complexity challenge* to include the lack of availability, ineffective needs-based accommodation, and poor accessibility for those managing multiple conditions across fragmented care sectors [38]. The complexity framework describes complexity as the number of diagnosed conditions (eg, multimorbidity), and also encompasses dimensions of other interconnected care needs such as biopsychosocial factors, sociopolitical factors, and the physical environment [39]. For example, Wagner’s Chronic Care Model provides key insights that focus on quality of life, function, and on disease control while tailoring treatment to the individual’s needs [40]. Our findings resonate with these studies by describing complexity in part because of multimorbidity (eg, relevant to the complexity of managing multiple, interacting conditions, and self-management concerns), complexity in terms of health care utilization (the lack of access, high cost, and readmission rate), and complexity because of psychosocial factors (mental health and other cultural factors). Furthermore, Wagner’s framework supports a collaborative, integrated care model that relies in part on the reorganization of health care *delivery system design* to support patients with CCN [40]. Identifying these needs and reorganizing care delivery will transform these gaps into practice opportunities that start to frame needs-based care for patients in an integrated model.

An integrated NP-led model of care focused specifically on patients with CCN would align with sustained calls for a common trajectory of multimorbidity and chronic disease management that focuses on patients from a more holistic and needs-based perspective [9,37]. Our findings suggest that a colocated model with a primary clinical contact can address the challenges faced by patients and clinicians in ambulatory care. Collaboration and coordination depend on an individual clinician who acts as a central coordinator of a patient’s needs as well as the needs of the interdisciplinary team to care for the patient. Other authors have described the need for *a connector* to *manage health*, someone for patients with CCN to rely on in times of critical need within the system [41]. Haggerty further suggests this individual should be one with the most comprehensive clinical knowledge of the patient. On the basis of our interviews, NPs are in this unique position to lead comprehensive and frequent management of complex needs [42]. Findings revealed that participants felt confident in the NP’s role to facilitate complex care within an integrated model. In fact, several participants described their reliance on the NP and integrated team to manage their needs, despite likely entrenched knowledge of traditional models of care delivery (eg, primary care and specialty care) and conventional leadership roles of medical providers (eg, physicians and nurses). In Canada, although NPs have been in practice since the early 1970s [43], role recognition outside of primary care [44] and funding [45] remain significant barriers to clinical practice, role familiarity, and scope expansion. Integrated colocated models of care are historically entrenched within primary care. A recent systematic review found 38 primary care articles that referred to colocated teams [46]. However, our findings suggest this level of care coordination is not consistent in either primary or specialty care for patients with CCN, thus creating gaps. To be clear, our intention is not to suggest a replacement to primary care but to detail findings that suggest a more step-down approach to ambulatory care for patients with CCN, particularly after an emergency department visit or hospitalization. Flexibility in building new models of care is required to position this type of unique model of complex care between traditional primary care and specialty care delivery.

Furthermore, this study identified the need for a comprehensive care approach within the model for nontraditional conditions such as specific mental health conditions (eg, anxiety and depression) in patients with CCN. And yet, recent evidence suggests this may be an oversimplification. Previous studies have suggested that closely linking physical and mental health in an integrated care model could inadvertently undermine the mental health treatment if physical management becomes privileged in care plan [47]. Along with others, we suggest embedding designated mental health providers within an integrated care model for patients with CCN to establish a therapeutic approach to care integration.

Finally, the identification of electronic health technologies that could be used to support innovative models of care through user-centered design is equally important for patients with often multiple CCN [48]. Both participant groups were interested in the idea of utilizing TM to support the needs of patients with CCN. Specifically, potential benefits included increasing accessibility to care services while providing a tool to improve self-management. Although this resonates with previous research on the value of TM for single chronic conditions [19-21,23,49], our findings suggest that TM needs to facilitate the management of more than one condition to be relevant within a colocated integrated model. These findings build on this literature, identifying features valuable to patients specifically with CCN, including the ability for patients to comment on specific readings or symptoms and face-to-face conferencing to reduce the need for in-person visits [50]. TM aims to support patients in the self-management of their condition(s) and improve communication and coordination within the circle of care [51,52]. In addition, developing a structured clinical workflow around TM-enabled triage will ensure that all team members, including patients, have clearly defined responsibilities within this novel integrated care model.

Our findings, along with previous research, suggest that TM could be successfully embedded in a novel integrated model of care, specifically for patients with CCN. We offer the following perspectives for clinicians, administrators, and policy makers to consider in developing integrated care models for patients with CCN:

1. A new model of integrated care is required to manage patients with CCN.
2. NPs are in a unique position to lead an integrated, ambulatory model of care for patients with CCN. NPs can facilitate frequent monitoring and coordination between the interdisciplinary team and across care sectors.
3. TM would be instrumental to support patients with CCN in integrated models of care.
4. TM can be managed by an RN, triaging and delegating when clinically indicated.
5. Key features of TM include routine monitoring metrics that patients with CCN already routinely measure (blood pressure, weight, and blood sugar) as well as direct text messaging, face-to-face video communication, and comment fields.

**Figure 1:**
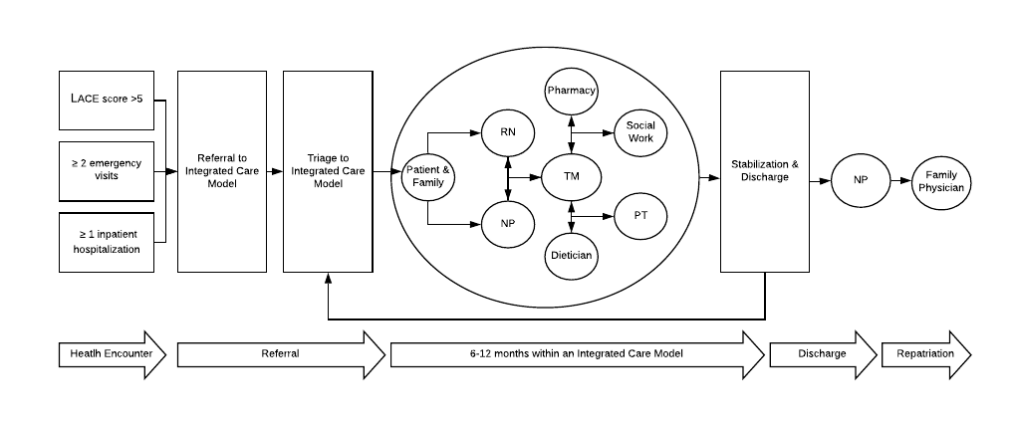
A care model map of a nurse practitioner–led integrated care model with an embedded telemonitoring system. LACE: Length of stay, Acuity of admission, Comorbidities, Emergency department visits; NP: nurse practitioner; PT: patient, RN: registered nurse; TM: telemonitoring.

Using the subthemes identified, an initial care model map was drafted of an NP-led integrated care model with an embedded TM system (Figure 1). Future research will explore the feasibility of implementation of TM within this type of integrated care model. Finally, a larger evaluation is necessary to determine if TM in this model can alter patient, process, or organizational outcomes, such as mitigating acute exacerbations in chronic conditions.

### Strengths and Limitations

An inductive qualitative description approach enabled researchers to obtain rich perspectives from a diverse group of participants. The interview guide was intentionally broad to capture an array of needs from multiple perspectives including on TM. However, due to time and resources, family members and caregivers were not specifically interviewed in this study, which may have limited the identification of complex care needs. In addition, this study was conducted at a single organization within a single health network, which could have limited the generalizability of the findings. However, this exploration of the needs of patients with CCN may provide valuable insights to other health care organizations across Canada looking to innovate and integrate health service delivery in ambulatory care. A mixed method study is now well underway to pilot the feasibility of embedding a mHealth-based TM system into this unique integrated model of care for patients with CCN. Future work will evaluate the components of integrated care in conjunction with the TM features necessary to meet the needs of patients with CCN.

### Conclusions

Although developing innovative models of care creates clear challenges within the currently entrenched health care service delivery models, the demand for change is only growing. NPs are in a unique position to lead integrated, colocated, and multidisciplinary teams with comprehensive and holistic approaches to person-centered chronic disease management. Patients are confident in NP-led teams managing complex chronic care and suggest they are in a position to address their needs from a central point of contact. Because of inadequate health care delivery, patients are seeking opportunities outside of traditional care delivery models to seek better experiences within the health care system. These participants are open to new technology, such as TM, to address the current gaps in care, such as lack of access, challenges related to their complexity, and/or multimorbidity and communication discontinuity. TM within NP-led integrated care models is an opportunity to facilitate frequent and actionable monitoring of patients with more than one condition. Finally, developing a structured clinical workflow around TM-enabled triage will ensure that all team members, including patients, have clearly defined responsibilities within this colocated, integrated model of care for patients with CCN.

## Appendix

Multimedia Appendix 1 Semistructured interview guide.

## Abbreviations

CCN: complex chronic needs
COPD: chronic obstructive pulmonary disease
CTM: care team members
HF: heart failure
LACE: Length of stay, Acuity of admission, Comorbidities, Emergency department visits
mHealth: mobile health
NP: nurse practitioner
PT: patient
RN: registered nurse
TM: telemonitoring

## References

[1] Marengoni A, Angleman S, Melis R, Mangialasche F, Karp A, Garmen A, Meinow B, Fratiglioni L (2011). Aging with multimorbidity: a systematic review of the literature. Ageing Res Rev.

[2] Skinner HG, Coffey R, Jones J, Heslin KC, Moy E (2016). The effects of multiple chronic conditions on hospitalization costs and utilization for ambulatory care sensitive conditions in the United States: a nationally representative cross-sectional study. BMC Health Serv Res.

[3] (2011). Canadian Institute for Health Information.

[4] Wodchis WP, Austin PC, Henry DA (2016). A 3-year study of high-cost users of health care. Can Med Assoc J.

[5] Muratov S, Lee J, Holbrook A, Paterson JM, Guertin JR, Mbuagbaw L, Gomes T, Khuu W, Pequeno P, Tarride J (2019). Unplanned index hospital admissions among new older high-cost health care users in Ontario: a population-based matched cohort study. CMAJ Open.

[6] Hajat C, Stein E (2018). The global burden of multiple chronic conditions: a narrative review. Prev Med Rep.

[7] Valentijn PP, Schepman SM, Opheij W, Bruijnzeels MA (2013). Understanding integrated care: a comprehensive conceptual framework based on the integrative functions of primary care. Int J Integr Care.

[8] Struckmann V, Leijten FR, van Ginneken E, Kraus M, Reiss M, Spranger A, Boland MR, Czypionka T, Busse R, Rutten-van Mölken M, SELFIE consortium (2018). Relevant models and elements of integrated care for multi-morbidity: results of a scoping review. Health Policy.

[9] Salisbury C, Man M, Bower P, Guthrie B, Chaplin K, Gaunt DM, Brookes S, Fitzpatrick B, Gardner C, Hollinghurst S, Lee V, McLeod J, Mann C, Moffat KR, Mercer SW (2018). Management of multimorbidity using a patient-centred care model: a pragmatic cluster-randomised trial of the 3D approach. Lancet.

[10] Barr V, Robinson S, Marin-Link B, Underhill L, Dotts A, Ravensdale D, Salivaras S (2003). The expanded Chronic Care Model: an integration of concepts and strategies from population health promotion and the Chronic Care Model. Hosp Q.

[11] Tinetti ME, Fried TR, Boyd CM (2012). Designing health care for the most common chronic condition--multimorbidity. J Am Med Assoc.

[12] Organisation for Economic Co-Operation and Development (2011). Health Reform: Meeting the Challenge of Ageing and Multiple Morbidities.

[13] Renders CM, Valk GD, Griffin SJ, Wagner EH, Van JT, Assendelft WJ (2001). Interventions to improve the management of diabetes in primary care, outpatient, and community settings: a systematic review. Diabetes Care.

[14] Kitsiou S, Paré G, Jaana M, Gerber B (2017). Effectiveness of mHealth interventions for patients with diabetes: an overview of systematic reviews. PLoS One.

[15] Seto E, Leonard KJ, Cafazzo JA, Barnsley J, Masino C, Ross HJ (2012). Perceptions and experiences of heart failure patients and clinicians on the use of mobile phone-based telemonitoring. J Med Internet Res.

[16] Gray CS, Miller D, Kuluski K, Cott C (2014). Tying eHealth tools to patient needs: exploring the use of eHealth for community-dwelling patients with complex chronic disease and disability. JMIR Res Protoc.

[17] He T, Liu X, Li Y, Wu Q, Liu M, Yuan H (2017). Remote home management for chronic kidney disease: a systematic review. J Telemed Telecare.

[18] Queirós A, Alvarelhão J, Cerqueira M, Silva A, Santos M, Rocha NP (2018). Remote care technology: a systematic review of reviews and meta-analyses. Technol.

[19] Paré G, Jaana M, Sicotte C (2007). Systematic review of home telemonitoring for chronic diseases: the evidence base. J Am Med Inform Assoc.

[20] Kitsiou S, Paré G, Jaana M (2015). Effects of home telemonitoring interventions on patients with chronic heart failure: an overview of systematic reviews. J Med Internet Res.

[21] Yun JE, Park JE, Park HY, Lee HY, Park DA (2018). Comparative effectiveness of telemonitoring versus usual care for heart failure: a systematic review and meta-analysis. J Card Fail.

[22] Cruz J, Brooks D, Marques A (2014). Home telemonitoring effectiveness in COPD: a systematic review. Int J Clin Pract.

[23] Seto E, Leonard KJ, Cafazzo JA, Barnsley J, Masino C, Ross HJ (2012). Mobile phone-based telemonitoring for heart failure management: a randomized controlled trial. J Med Internet Res.

[24] Paré G, Moqadem K, Pineau G, St-Hilaire C (2010). Clinical effects of home telemonitoring in the context of diabetes, asthma, heart failure and hypertension: a systematic review. J Med Internet Res.

[25] Mochari-Greenberger H, Vue L, Luka A, Peters A, Pande RL (2016). A tele-behavioral health intervention to reduce depression, anxiety, and stress and improve diabetes self-management. Telemed J E Health.

[26] Koehler F, Koehler K, Deckwart O, Prescher S, Wegscheider K, Kirwan B, Winkler S, Vettorazzi E, Bruch L, Oeff M, Zugck C, Doerr G, Naegele H, Störk S, Butter C, Sechtem U, Angermann C, Gola G, Prondzinsky R, Edelmann F, Spethmann S, Schellong SM, Schulze PC, Bauersachs J, Wellge B, Schoebel C, Tajsic M, Dreger H, Anker SD, Stangl K (2018). Efficacy of telemedical interventional management in patients with heart failure (TIM-HF2): a randomised, controlled, parallel-group, unmasked trial. Lancet.

[27] Ong MK, Romano PS, Edgington S, Aronow HU, Auerbach AD, Black JT, De Marco T, Escarce JJ, Evangelista LS, Hanna B, Ganiats TG, Greenberg BH, Greenfield S, Kaplan SH, Kimchi A, Liu H, Lombardo D, Mangione CM, Sadeghi B, Sadeghi B, Sarrafzadeh M, Tong K, Fonarow GC, Better Effectiveness After Transition–Heart Failure (BEAT-HF) Research Group (2016). Effectiveness of remote patient monitoring after discharge of hospitalized patients with heart failure: the better effectiveness after transition -- heart failure (BEAT-HF) randomized clinical trial. JAMA Intern Med.

[28] Smith SM, Soubhi H, Fortin M, Hudon C, O'Dowd T (2012). Managing patients with multimorbidity: systematic review of interventions in primary care and community settings. Br Med J.

[29] Bradshaw C, Atkinson S, Doody O (2017). Employing a qualitative description approach in health care research. Glob Qual Nurs Res.

[30] Sandelowski M (2000). Whatever happened to qualitative description?. Res Nurs Health.

[31] Sandelowski M (2010). What's in a name? Qualitative description revisited. Res Nurs Health.

[32] Gray CA, Gill A, Khan AI, Hans P, Kuluski K, Cott C (2016). The electronic Patient Reported Outcome (ePRO) Tool: testing usability and feasibility of a mobile app for patients with complex chronic disease and disability in primary care settings. Int J Integr Care.

[33] Naganathan G, Kuluski K, Gill A, Jaakkimainen L, Upshur R, Wodchis WP (2016). Perceived value of support for older adults coping with multi-morbidity: patient, informal care-giver and family physician perspectives. Ageing Soc.

[34] Saunders B, Sim J, Kingstone T, Baker S, Waterfield J, Bartlam B, Burroughs H, Jinks C (2018). Saturation in qualitative research: exploring its conceptualization and operationalization. Qual Quant.

[35] Birt L, Scott S, Cavers D, Campbell C, Walter F (2016). Member checking: a tool to enhance trustworthiness or merely a nod to validation?. Qual Health Res.

[36] Ho JW, Kuluski K, Im J (2017). 'It's a fight to get anything you need' - Accessing care in the community from the perspectives of people with multimorbidity. Health Expect.

[37] Rosella L, Kornas K (2018). Putting a population health lens to multimorbidity in Ontario. Healthc Q.

[38] Song HJ, Dennis S, Levesque J, Harris MF (2019). What matters to people with chronic conditions when accessing care in Australian general practice? A qualitative study of patient, carer, and provider perspectives. BMC Fam Pract.

[39] Schaink AK, Kuluski K, Lyons RF, Fortin M, Jadad AR, Upshur R, Wodchis WP (2012). A scoping review and thematic classification of patient complexity: offering a unifying framework. J Comorb.

[40] Wagner E (1998). Chronic disease management: what will it take to improve care for chronic illness?. Eff Clin Pract.

[41] Kuluski K, Peckham A, Gill A, Gagnon D, Wong-Cornall C, McKillop A, Parsons J, Sheridan N (2019). What is important to older people with multimorbidity and their caregivers? Identifying attributes of person centered care from the user perspective. Int J Integr Care.

[42] Haggerty JL (2012). Ordering the chaos for patients with multimorbidity. Br Med J.

[43] Kaasalainen S, Martin-Misener R, Kilpatrick K, Harbman P, Bryant-Lukosius D, Donald F, Carter N, DiCenso A (2010). A historical overview of the development of advanced practice nursing roles in Canada. Nurs Leadersh (Tor Ont).

[44] Delvin ME, Braithwaite S, Plazas PC (2018). Canadian nurse practitioner's quest for identity: a philosophical perspective. Int J Nurs Sci.

[45] (2016). Canadian Nurses Association.

[46] Saint-Pierre C, Herskovic V, Sepúlveda M (2018). Multidisciplinary collaboration in primary care: a systematic review. Fam Pract.

[47] Knowles SE, Chew-Graham C, Adeyemi I, Coupe N, Coventry PA (2015). Managing depression in people with multimorbidity: a qualitative evaluation of an integrated collaborative care model. BMC Fam Pract.

[48] Gray CS, Mercer S, Palen T, McKinstry B, Hendry A (2016). eHealth advances in support of people with complex care needs: case examples from Canada, Scotland and the US. Healthc Q.

[49] Seto E, Leonard KJ, Cafazzo JA, Masino C, Barnsley J, Ross HJ (2011). Mobile phone-based remote patient monitoring improves heart failure management and outcomes: a randomized controlled trial. J Am Coll Cardiol.

[50] Mallow JA, Petitte T, Narsavage G, Barnes E, Theeke E, Mallow BK, Theeke LA (2016). The use of video conferencing for persons with chronic conditions: a systematic review. Ehealth Telecommun Syst Netw.

[51] Ong SW, Jassal SV, Miller JA, Porter EC, Cafazzo JA, Seto E, Thorpe KE, Logan AG (2016). Integrating a smartphone-based self-management system into usual care of advanced CKD. Clin J Am Soc Nephrol.

[52] Aminuddin HB, Jiao N, Jiang Y, Hong J, Wang W (2019). Effectiveness of smartphone-based self-management interventions on self-efficacy, self-care activities, health-related quality of life and clinical outcomes in patients with type 2 diabetes: A systematic review and meta-analysis. Int J Nurs Stud.

