# Correction: Exploring an Innovative Care Model and Telemonitoring for the Management of Patients with Complex Chronic Needs: Qualitative Description Study

**DOI:** 10.2196/53832

**Published:** 2023-10-25

**Authors:** Kayleigh Gordon, Carolyn Steele Gray, Katie N Dainty, Jane DeLacy, Patrick Ware, Emily Seto

**Affiliations:** 1 University of Toronto Toronto, ON Canada; 2 Centre for Global eHealth Innovation Techna Institute University Health Network Toronto, ON Canada; 3 Bridgepoint Collaboratory for Research and Innovation Lunenfeld-Tanenbaum Research Institute Sinai Health System Toronto, ON Canada; 4 North York General Hospital North York, ON Canada; 5 William Osler Health System Brampton, ON Canada

In “Exploring an Innovative Care Model and Telemonitoring for the Management of Patients with Complex Chronic Needs: Qualitative Description Study” (JMIR Nursing 2020;3(1):e15691), the authors made one addition.

An Acknowledgments section has been added that reads as follows:

This research was made possible by the funding support from a Canadian Institutes of Health Research Personalized Health Catalyst Grant (Funding Reference Number 155443).

The correction will appear in the online version of the paper on the JMIR Publications website on October 25, 2023 together with the publication of this correction notice. Because this was made after submission to PubMed, PubMed Central, and other full-text repositories, the corrected article has also been resubmitted to those repositories.

