# Supplementary material for: Exploring an Innovative Care Model and Telemonitoring for the Management of Patients With Complex Chronic Needs: Qualitative Description Study
Source: JMIR Nurs. 2020 Mar 6;3(1):e15691. doi: 10.2196/15691 (PMC8279442; doi:10.2196/15691)
Supplement: Multimedia Appendix 1 [file nursing_v3i1e15691_app1.docx]

1. Tell me about your health?
2. Do you have any challenges managing your health or current conditions?
   1. If so, how do they affect your health overall?
3. What can you think of that would help you manage your health or conditions better?
   1. What specific aspects of your conditions do you need help with?
   2. How do you manage the signs/symptoms of your condition(s)?
   3. How could this help be delivered to you?
   4. Is there a technology that would help you manage your conditions?
   5. We are also interested how men and women manage their conditions.
      1. In your opinion, do you think men and women need different kinds of help to manage their conditions?
      2. If so, how would this type of help differ? Why or why not?
4. What do you think would help your healthcare providers manage your condition(s) better?

We will be looking at new ways to improve healthcare. We would like to create a new clinic at William Osler Health System that is made up of different kinds of health care providers working together.

1. What do you think about a new clinic with different kinds of health providers at one site?
   1. Probe: Do you have any other doctors or clinics that work in a similar way? If yes, please describe this clinic/process.
2. Is there anything that could be improved or you dislike your experience with seeking care?
3. What type of setup would work best for you when you come in for appointments?
4. How do you think your clinicians should work together?
   1. Probe: Say you have a question or need help from a health care provider, who do you want to contact about your care (i.e. a nurse practitioner?)
   2. Probe: Who would you want available to talk to?
      1. Is the role important to you? A Nurse/Doctor/Pharmacist?
5. Do you monitor any physiological measures at home right now?
   1. Probe: Blood pressure, Blood sugar, etc.?

We are interested in using a new type of technology within the clinic called telemonitoring. Telemonitoring is a way for clinicians to monitor your health in-between appointments. Telemonitoring can monitor your chronic condition(s) at home, for example taking your blood pressure or answering questions about your symptoms. All this information is sent to your clinicians.

1. Do you think a telemonitoring system could be helpful for you, in managing multiple chronic conditions? How so?
   1. Probe: Could you see yourself using this at home? If so, in what way?
   2. Probe: What would work for you? Why?
   3. Probe: Do you see any challenges with it?
   4. Probe: Do you foresee any specific challenges to being a man or a woman using a system like this? Are there any road-blocks? Why or why not?
2. Do you see have any ideas of features we could develop that would be helpful to you? Why or why not?
   1. Probe: What would be the most useful things it could do for you?
   2. Probe: What kinds of things would you add in?
3. Is this something you could use for a long time?
   1. Probe: How long would you use it for?

Thank you for your time today. The information you shared is

very important to the team and to helping us continue to improve patient care.

If we have any follow up questions, may we contact you?

In the future, would you be interested in participating in actually using TM when we get to that stage?

1. Is there anything else you would like to discuss before we end the interview?
